# Born-Blindness Preventable

**Published:** 1890-08

**Authors:** 


### BORN-BLINDNESS PREVENTABLE.

Statistics taken from the reports of Fuchs, Magnas, Howe and the committee of the Ophthalmological Society of the United Kingdom, show that at least thirty per cent. of all blindness in Europe and in this country is caused by preventable disease at birth. The census of 1880 gives a total of about fifty thousand blind in the United States. Of these, at least fifteen thousand have been blind from birth. And yet, this disease is well nigh absolutely preventable, and in its incipiency, easily curable. This statement is borne out by facts, as will be seen by reference to the reports of the large lying-in hospitals, where the methods of prevention have been in operation. After these means were put into operation, there was practically an entire disappearance of the disease. The method consists in wiping the face and lids clean and dry immediately after the umbilical cord is tied. The lids are then opened, and one or two drops of a two per cent. solution of nitrate of silver are instilled. Except in premature children the reaction from this treatment is very slight.

It is obvious that our first duty is to arouse our teachers and writers on obstetrics, to the necessity of instructing their pupils as to the proper care of the eyes of the child from the very instant of its birth. Let them be instructed to wash the eyes with some antiseptic solution, and examine into their condition at each visit, for at least a week.

